# The grapevine gene nomenclature system

**DOI:** 10.1186/1471-2164-15-1077

**Published:** 2014-12-06

**Authors:** Jérôme Grimplet, Anne-Françoise Adam-Blondon, Pierre-François Bert, Oliver Bitz, Dario Cantu, Christopher Davies, Serge Delrot, Mario Pezzotti, Stéphane Rombauts, Grant R Cramer

**Affiliations:** Instituto de Ciencias de la Vid y del Vino (CSIC, Universidad de La Rioja, Gobierno de La Rioja), Logroño, 26006 Spain; INRA, Unité de Recherche Génomique-Info (URGI), Route de Saint Cyr, 78026 Versailles, France; Université de Bordeaux, ISVV, EGFV, UMR 1287, F-33140 Villenave d’Ornon, France; Department of Grapevine Breeding, Geisenheim University, 65366 Geisenheim, Germany; Department of Viticulture and Enology, University of California, Davis, CA 95616 USA; CSIRO Plant Industry, Waite Campus, Urrbrae, Mitcham, 5064 SA Australia; Department of Biotechnology, Università degli Studi di Verona, 37134 Verona, Italy; Department of Plant Systems Biology, Vlaams Instituut voor Biotechnologie, B-9052 Ghent, Belgium; Department of Plant Biotechnology and Bioinformatics, Ghent University, B-9052 Ghent, Belgium; Department of Biochemistry and Molecular Biology, University of Nevada, Reno, NV 89557 USA

## Abstract

**Background:**

Grapevine (*Vitis vinifera* L.) is one of the most important fruit crops in the world and serves as a valuable model for fruit development in woody species. A major breakthrough in grapevine genomics was achieved in 2007 with the sequencing of the *Vitis vinifera* cv. PN40024 genome. Subsequently, data on structural and functional characterization of grape genes accumulated exponentially. To better exploit the results obtained by the international community, we think that a coordinated nomenclature for gene naming in species with sequenced genomes is essential. It will pave the way for the accumulation of functional data that will enable effective scientific discussion and discovery. The exploitation of data that were generated independently of the genome release is hampered by their heterogeneous nature and by often incompatible and decentralized storage. Classically, large amounts of data describing gene functions are only available in printed articles and therefore remain hardly accessible for automatic text mining. On the other hand, high throughput “Omics” data are typically stored in public repositories, but should be arranged in compendia to better contribute to the annotation and functional characterization of the genes.

**Results:**

With the objective of providing a high quality and highly accessible annotation of grapevine genes, the International Grapevine Genome Project (IGGP) commissioned an international Super-Nomenclature Committee for Grape Gene Annotation (sNCGGa) to coordinate the effort of experts to annotate the grapevine genes. The goal of the committee is to provide a standard nomenclature for locus identifiers and to define conventions for a gene naming system in this paper.

**Conclusions:**

Learning from similar initiatives in other plant species such as *Arabidopsis*, rice and tomato, a versatile nomenclature system has been developed in anticipation of future genomic developments and annotation issues. The sNCGGa’s first outreach to the grape community has been focused on implementing recommended guidelines for the expert annotators by: (i) providing a common annotation platform that enables community-based gene curation, (ii) developing a gene nomenclature scheme reflecting the biological features of gene products that is consistent with that used in other organisms in order to facilitate comparative analyses.

## Background

As for many other major model plant species, the release of the grapevine genome in 2007 [1] led to a rapid accumulation of “Omics”-scale data and a burst of high-throughput studies. In 2010, the *V. vinifera* cv. PN40024 genome sequence was updated from 8X to 12X coverage [2] and is, to date, the reference genome for *V. vinifera*. The gene models and their putative functions have been automatically predicted from the genome sequence and have been used in many functional studies. The results from these published studies were deposited in general-purpose gene databases such as NCBI, but also in other independent repositories. These data are a highly informative resource to help curate the automatic prediction. Another resource, consisting of manually curated gene families associated with heterogeneous levels of functional evidence is also growing rapidly [3–6] but lacks a central storage system allowing coordination of gene nomenclature. Previous important efforts have been made in the past to curate the automated functional annotation [7]. These data are publicly available, but are not well integrated into major genomic databases such as NCBI and EBI.

To streamline the new nomenclature initiative from the sNCGGa, a set of directives, addressing the most important issues, has to be provided to allow a better integration of the various, diverse resources into an improved global annotation of the grapevine genome, both at the structural and functional levels. These directives are aimed at facilitating exchanges between international genomic repositories to assist the analysis of gene experimental functional data and comparisons with other species.

In addition to the sequencing of the nearly homozygous PN40024 genome, other genomic resources for *V. vinifera,* and related species, continue to be generated, including the sequencing of the genomes of other varieties [8–10], EST sequencing, integrated genetic maps, and the whole genome re-sequencing for polymorphism discovery of other *Vitis* varieties and species [11]. The EST and genome resources have permitted the design of a wide variety of microarrays for large-scale mRNA expression profiling studies (for example: [12]), but microarrays are being replaced by RNA-seq (for example: [9]). A majority of the expression data are maintained in the PLEXdb database [13]. However, heterogeneity in the design of the microarray platforms, both in terms of the version of the annotation and in technical design, requires considerable bioinformatic effort to identify the probes or probesets corresponding to a unique gene. Besides, the assembly of the genome of other varieties [9, 10] and the elucidation of their transcriptomes [14], produce varietal specific sets of genes that will have to be traced. These under-exploited resources can be better used to improve the annotation of the reference genome.

The availability of the annotated genome sequence also facilitates the identification of proteins resulting from mass spectrometry analyses and increases the effectiveness of high throughput proteomics studies in grapevine [15]. Proteomic analyses have been used to characterize differential expression of proteins underlying diverse aspects of grapevine physiology in the berry or vegetative tissues [15, 16]. Furthermore, information acquired from these studies on the potential functional role of the genes coding for these proteins would benefit gene annotation curation. Conversely, the continuous improved annotation will impact favourably on expression and proteomics analyses, provided this annotation remains easily accessible.

To achieve our goals, a network of annotation experts with a clearly defined strategy and *modus operandi* is needed. From the several plant genomes sequenced in recent years, only *Arabidopsis* has really benefited from a comprehensive monitoring and a real refinement of data generated automatically. This was mainly because of the existing large scientific community, supplied with significant financial support from granting agencies, allowing the development of resources such as TAIR [17]. Rice [18] and tomato [19] are at an intermediate level; their data curation structures have been established. The herein proposed directives have been inspired by the sets of rules for gene nomenclature that are available for *Arabidopsis* [20], rice [21], *Medicago* [22] and tomato [23].

The grapevine genomics community at large is mostly structured around the International Grape Genome Program (IGGP; http://www.vitaceae.org) whose mission is to facilitate the networking of grapevine researchers in order to develop common and publicly available resources. These resources facilitate the elucidation of the genetic and molecular basis of biological processes in *Vitis* and should lead to a more efficient exploitation of the *Vitis* biological resources for the development of new cultivars and clones that have improved quality and reduced economic and environmental costs. It may also allow for more efficient vineyard management.

**Figure 1:**
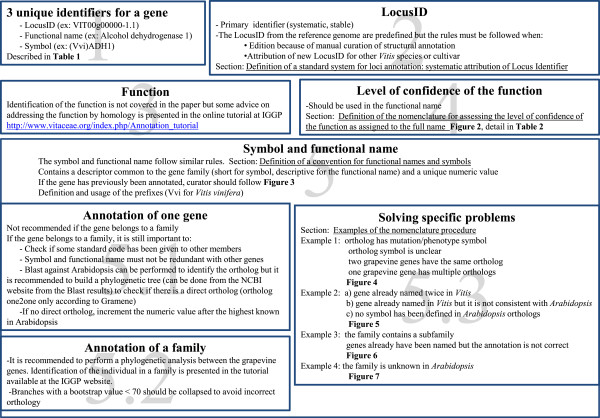
**Summary of the point raised in each of the sections.**

It is therefore the IGGP’s objective to provide a common platform for continuous improvement of the annotation of grapevine genes. This objective will be coordinated by the Supernomenclature Committee for Grapevine Gene Annotation (sNCGGa), and was supported by the Grape Research Coordination Network (funded by the United States National Science Foundation in the USA). The first milestone presented here is the development of a standardized protocol for gene naming, with names that have to be unique, consistent with other plant models and sustainable. This report proposes guidelines for the nomenclature of the genes from the latest version of the gene structural annotation, promoted by the COST (European Cooperation in Science and Technology) ACTION FA1106 (funded by the European Union), and performed on the assembly (V2) of the scaffold from the 12X version of the reference genome performed in a collaboration between the Institut National de la Recherche Agronomique (INRA) and the Istituto di Genomica Applicata (IGA). The automatic annotation of the genes was performed with the Eugene software [24] at the Vlaams Instituut voor Biotechnologie (VIB) and released through the ORCAE website that will be used for community annotation [25]. The important points addressed in each section of the manuscript to help gene annotators to address specific issues that they may encounter are highlighted in Figure 1.

## Results & discussion

### Nomenclature and definition of the gene naming system and convention

There are three main categories of nomenclature that need to be addressed for each gene (Table 1). In the first place, the Locus Identifier (Locus ID), will represent the unique identifier of the gene in the genome. This identifier is not intended to be related to a physical position on the chromosome. The second and the third places correspond to the Full Name and the Symbol, respectively, and refer to the description of the functional role of the protein encoded by the gene. The Symbol is a short abbreviation of the full name. To deal with pre-existing naming schemes we propose to add synonyms. These correspond to other types of names that have been encountered in the literature; they can be symbols or full names.

**Table 1:** **Brief definition and example of the main elements of the gene nomenclature**

Elements	Locus ID	Full name	Symbol	Synonyms
Example	Vitvi18g12230	(*Vitis vinifera*) Alcohol dehydrogenase 1	(Vvi)ADH1	GV-ADH1 aldehyde reductase, ethanol dehydrogenase
Description	Genome localization	Relatively descriptive function, include the level of curation (see Figure 2)	Concise (3–10 characters), should be descriptive of function when possible	Any known synonyms

#### Definition of a standard system for loci annotation: systematic attribution of locus identifier

A Locus-ID will be assigned to all genetic objects having a unique position on the genome. This Locus ID provides a unique identifier initially provided after automatic annotation to a specific object along the genome. Locus-IDs under no circumstances can be re-used, but objects, like genes, can be changed when corrected. Initially, the numbering will be incremental along the chromosomes. With updates of the assembly, and the moving of unanchored contigs from chromosome “00” to their real location, new Locus-IDs will be created in series, as detailed in the “numeric code” section, replacing the chromosome “00”-related Locus-IDs. Merging (concatenating) gene models will follow the same rules, with the difference that one of the Locus-ID’s will be discarded. In the case of splitting gene models, a new Locus-ID will be created and attributed to the new gene model. As such, Locus-IDs should not be seen as positional and derived products; however, transcripts and proteins will remain linked to these Locus-IDs. These rules can be virtually applied to any objects that are absent from the reference genome, such as genes that are only identified in other cultivars or *Vitis* species. Non-reference genes can then be referenced with their chromosome number (or “00” if unknown) and a numeric code can be stored in the ORCAE platform [25] that will be used for community annotation.

Taking into account previous experiences acquired through the previous grapevine locus ID schemes [26] and structures defined in other species, an ID containing the following elements was retained: Taxonomy ID/Chromosome number/Object type/Numeric code/Sequence variant/Version.

Each element separated by a slash has a specific function as described below.

##### Taxonomy ID

For the reference genome of the *V. vinifera* var. PN40024, it was decided to follow the species abbreviation list that exists at UniProt [27], and the Supernomenclature Committee considered using this five-digit code for *V. vinifera* ,‘VITVI’ (three letters for the genus and two for the species). This abbreviation is widely used in UniProt for gene abbreviation, but more rarely for locus name, but it was considered the best long-term solution. Other important plant species have their own strategies. In tomato (*Solanum lycopersicum)*, a five-letter code is used with two letters for the genus and three for the species; SOLYC instead of SOLLC as recommended at UniProt. Note that the *Brassica* community also uses a three-letter code [28], while most of the other species use two letter codes. For other *Vitis* species, the most widely occurring *Vitis* species already appear in the UniProt species list and this abbreviation should be used. Prefixes for other species must include the three letters ‘VIT’ and the code defined by the *Vitis* International Variety (VIV) Catalogue [29], for example the code for *Vitis berlandieri* should be VITVBR, with six letters. This code must be utilized when registering new genome sequencing of a *Vitis* species. No reference should be made to the cultivar in the taxonomy ID, which should be done in the sequence variant section.

##### Chromosome number

The second item refers to the number of the chromosome to which the gene is predicted to be localized. The chromosome number is attributed as defined by the IGGP and ranges from 00 to 19. The chromosome “00” corresponds to an assembly, in a random order, of scaffolds that could not be positioned yet on the chromosomes.

##### Object type

The third item represents the type of object corresponding to the molecular entity: **g** for gene; **t** for protein coding transcript; **p** for protein; **nc** for non-coding; **tr** for transfer RNA; **te** for transposable element; **rr** for ribosomal RNA; **mi** for microRNA; **ps** for pseudogene; **si** for small interfering RNA; **sn** for small nuclear RNA. Initially and before curation, the “Object types” referring to the DNA structure are labeled with the “**g**” code when referring to the locus, the “**t**” code when referring to the nucleic acid coding sequence of the transcript and the “**p**” code when referring to the amino acid sequence of the protein.

##### Numeric code

The numeric code includes five digits that are initially defined in sequential order of the genes along a chromosome in ascending order from the telomere of the short arm (north side) to the telomere of the long arm (south side). In other species, it was decided to leave a gap between genes to allow the addition of further genes if new information was discovered. In *Arabidopsis* for instance, with a similar five-digit code, the gene IDs were numbered with an increment of 10 to allow room in-between currently annotated genes. In *Arabidopsis,* known gaps in the DNA sequence were assigned 200 ‘spare’ identifiers per 100 Kb of gap [20]. In rice [21], a seven-digit code was used and genes were assigned in increments of 100. In tomato, a six-digit code was used and genes were assigned in increments of 10. In the *Vitis* Locus ID, because further improvements of the assembly are expected, we decided that no gaps would be left between the numeric codes of the genes (increments +1). If new objects have to be defined in the future, the next available number will be allocated as Locus-ID. Indeed, this means that after future rounds of improvement of this annotation the ID number will not reliably reflect the gene order along the chromosome. However, we think that this method presents several advantages. Given that the grapevine genome is still a work in progress with many unanchored scaffolds and whole regions with unsecure orientations, we can anticipate that scaffolds will be inserted or re-oriented and that the chosen numbering method will not lead to the risk of running out of numbers in the case that the gaps between two genes are larger than foreseen. Such an event will not impact the nomenclature; even if it involves chromosome changes, the old Locus ID will be stored as a synonym and a new Locus ID will be allocated, while in the case of a change of scaffold orientation, nothing would change. With a length of 5 digits for all the objects per chromosome (up to 99,999), the risk of running out of numbers is very low. The ORCAE platform [25] being used by the grapevine community can automatically handle any changes to ID numbers, decreasing the risk of errors.

##### Sequence variant

This segment, which shall be preceded by a hyphen, will be used to discriminate molecular variants (allele, splice variant) that map to the same locus. The code can be numeric or alphabetic (e.g. for cultivar-specific polymorphism). If no allelic variant is present, one should refer to the primary sequence from the reference genome. Note that there would not be any cultivar-specific terms in the reference genome, these terms would be addressed in the species’ genomes. The splice variant is used only for object types “t” or “p”.

The choice of numeric or alphabetic naming of the section (allele, splice variant, cultivar etc.) is left to the authors’ discretion but it should be as concise as possible. As an example, it was identified that in the cultivar Tempranillo (abbreviated by the authors tp) that allele A produces mRNAs of splice form 1, 2, and 3; allele B produces mRNAs of splice form 1, 2, and 4; and Allele C produces mRNAs of splice form 1, 2, and 3. The sequence/splice variants as described above should be the following: −a1, −a2, −a3, −b1, −b2, −b4, −c1, −c2, −c3, or -tpa1, −tpa2, −tpa3, −tpb1, −tpb2, −tpb4, −tpc1, −tpc2, −tpc3, if the cultivar is mentioned. Authors must make sure that the code for the splice variant that they are defining is unique.

##### Version

Any modification (addition, deletion) of any number of nucleotides, of the structural annotation of a gene will result in incrementing (+1) the version number. Version numbers are appended at the end of the locus ID, separated by a dot. If omitted, the most recent version of the gene model is implied. Versions are used when the modifications do not require Locus-ID change.

#### Definition of the nomenclature for assessing the level of confidence of the function as assigned to the full name

**Figure 2:**
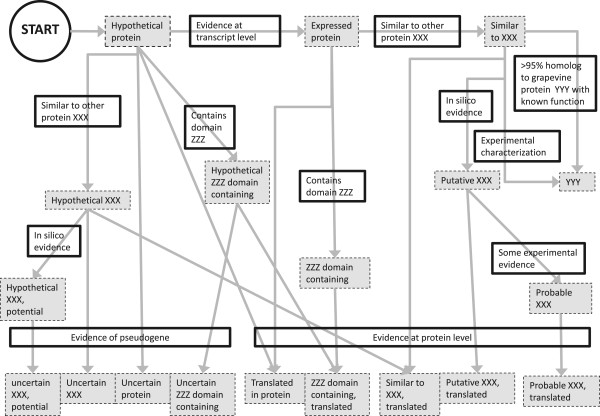
**Decision tree of rules for classifying sequences according to the level of evidence for its function.**

A guideline for defining the level of confidence of the annotation is presented in Figure 2. It is largely inspired by the guidelines proposed for the annotation of the rice [21] and tomato [23] genomes. Given that information obtained from experimental evidence is scarce in *Vitis*, it seems sensible to divide all loci into (i) those with defined, confirmed function (confirmed through biochemical characterization of the corresponding protein or the characterization of a mutant), (ii) those defined only by sequence similarity (‘putative names”) and (iii) genes of unknown function (including those with no match). Given the relative paucity of functional data available for grape it might be dangerous to suggest a “definitive” full name for a gene whose function has not been experimentally proven. On the other hand, not considering *in silico* inferred function would hide highly valuable information for hypothesis-driven experiments. We propose a set of guidelines that satisfy these considerations and the recommendations of UniProt in terms of the degree of proof that defines the different levels of quality of the functional annotation [30]. Definition of the terms from Figure 2 is presented in Table 2. *In silico* evidence*, experimental characterization* and *some experimental evidenc*e should lead to the assignment of the GO annotation and the GO field in the ORCAE database should be edited complying with the Evidence Codes for the Gene Ontology (GO) [31].

**Table 2:** **Definition of the level of curation terms**

Value	Definition
Hypothetical protein	Allocated to each locus at the beginning of the process, meaning that the gene codes for a protein, for which no information regarding its function or actual existence is known. It should be removed only when existence of transcript is proven.
Expressed	Replaces “hypothetical” if existence of transcripts has been proven through expression data (proof of existence of RNA(s): RT-PCR, EST, RNA-seq, Northern blots, microarrays, etc.). The next step is to determine if similarity with sequences in other species can be observed.
ZZZ domain containing	Allocated if by comparison with other sequences or by performing a domain analysis, the highest level of information on the coding protein is the presence of a given domain ZZZ.
Similar to	Indicates that the existence of a protein is probable because a minimal level of similarity with a protein from a plant species was met. An e-value of e-20 is considered to be a reasonable cut-off or to have at least 30% identity for at least 80 contiguous amino acids, which places it into the “safe zone” as defined by [32]. The gene is labelled here as “similar to XXX”, with “XXX” being the homologous protein from another species.
YYY	If the gene has been experimentally characterized and named YYY or if there is >*95% identical amino acids on the whole sequence to a grapevine protein YYY with a known function*, then the label should be the value “YYY” that corresponds to a gene whose function has been discovered and characterized in the *Vitis* Genus.
Putative	Derived from *in silico evidence* on function, indicates that there is some logical or conclusive evidence that the given annotation could apply. This non-experimental qualifier is often used to present results from protein sequence analysis software, which are only annotated if the result makes sense in the biological context of a given protein. A typical example is the annotation of N-glycosylation sites in secreted proteins.
Probable	Indicates stronger evidence than the qualifier “putative” on function. This qualifier implies that there must be at least *some experimental evidence*, which indicates that the information is expected to be found in the natural environment of a protein.
Uncertain	Indicates that the existence of the protein is unsure and that there is evidence that the sequence corresponds to a *pseudogen*e.
Translated	Is acquired when experimental *evidence at the protein level* indicates that there is clear proof of the existence of the protein. The criteria include partial or complete Edman sequencing, clear identification by mass spectrometry, X-ray or NMR structure, good quality protein-protein interaction or detection of the protein by antibodies.

#### Definition of a convention for functional names and symbols

The adoption of a common nomenclature across diverse organisms facilitates structural, functional, and evolutionary comparisons of genes and genetic variation. From the onset of genetic research, genes were often named referring to the mutant the genes could be linked to. This is not only true for plants, but this gene-naming scheme can hardly be maintained across many species or is sometimes confusing or even misleading when looking deeper at the evidence compiled using cutting edge technologies. Indeed, most of the early gene names and symbols describing visible phenotypes provided by the earliest evidence for the existence of a gene might not have the same effect or worse more genes that lead to a certain phenotype would end up with related name while being completely different. In grapevine, there is much less mutational data than in *Arabidopsis*, and only a few genes were named after a phenotype. However, the naming system should be developed to be flexible enough to cope with the expansion of data that will be produced in the future, including from yet to be invented technology. Therefore the goal should be a system where both the full name and the symbol are composed by a descriptive (full name) and/or a short (symbol) name referring to the function of the coding protein and a number to discriminate the isoform. In rice, this later number is known as the locus designator and indicates the chronological order in which a particular gene or gene family member was identified [21]. In grapevine, the function of most genes is in the large majority inferred by sequence similarity. The ‘guilt-by-association’ approach, however, presents problems when a single-copy, well-characterized gene from one plant corresponds to multiple grapevine paralogs. In this case, a consistent individual numbering system in grapevine needs to be put in place. Another issue raises when, through independent studies carried out by different authors, multiple names and symbols were given to genes that converge to a single locus in grapevine. It is also very common for enzymes to be represented by different synonyms for the same function. The aim of the nomenclature system is to state on rules where only one full name and one symbol, consistent with each other, will be attributed and where all the other known names will be considered as synonyms. Rules for the attribution of both the main name and the numbering of the members of gene families are described below. When naming enzymes, the use of the Enzyme Commission nomenclature (EC) for the primary name should be preferred and when possible, a bibliographic reference for the synonym should be stored in ORCAE (doi, Pubmed ID…). Names corresponding to mutant phenotype should be used when a mutant is available with the name describing some aspect of the corresponding phenotype. Names corresponding to gene product should be used regardless of the availability of a mutant when the symbol describes some aspect of gene structure or function.

**Figure 3:**
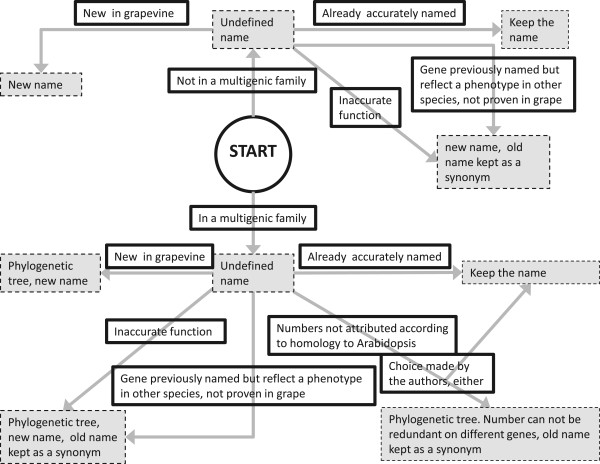
**Decision tree on the naming or possible renaming procedure of a gene.**

The gene symbol should consist of two to five letters if possible and the corresponding locus designator consisting of one to three digits. In *Brassicaceae*, the gene symbol can have up to six digits. In *Arabidopsis* and rice the use of species-specific prefixes (At, Os) for the symbol and the full name in the official name is discouraged because of redundancy with species information already known elsewhere (in the Locus ID, for example), the same shall apply for *Vitis*. However, it could be added when specifically referring to the *Vitis* gene in publications, with the *vinifera* prefix being Vvi and the other prefixes as shown in the VIV catalogue [29]. Although *Vitis vinifera* genes were named with the vv (or Vv) prefix, this creates confusion with the bacteria *Vibrio vulnificus*, whose genome was published before the grapevine and “locked” the vv prefix into major databases. A two-letter code is also too short for discrimination between *Vitis* species. The intention of this paper is to strive to a consistent naming scheme that would avoid redundancy and confusion within and across gene families. When a mutant phenotype exists in *Vitis,* the root of the full name and the symbol will refer to it, else it is recommended to use when possible the same symbol as the corresponding gene family in the model plant Arabidopsis to facilitate cross-species comparisons since it is the best annotated plant to date. Bearing these crucial rules in mind, several strategies can be followed for the numbering of the members of a gene family. It is recommended to use numbers based on phylogenetic or ‘guilt-by-association’, homology based approaches although we recognize that phylogenetic trees may evolve as more species are sequenced in the future and that the functional information of such numbering may therefore be less relevant after several years, specifically when the gene belongs to a large family, alternative can be used: keep historical names when they do exist, numbering in a chronological order of discovery and random numbering. Use of the position on the chromosome is not recommended because it will be misleading when new genes in the family are found or segments of the genome are rearranged.If an author plans to change or to update a name, we provide a summarizing decision tree in Figure 3, which we hope will allow one to evaluate what necessary steps to take that will lead to a appropriate naming. The next paragraphs give some case studies and recommendations for gene naming based on a phylogenetic approach.

### Gene naming based on phylogenetic trees

In order to provide a reproducible phylogenetic tree, it is recommended to follow the instructions on homology determination provided by Gramene [33] (the method was published in [34]). Only orthologs one2one should be considered when allocating the *Arabidopsis*-like name to the *Vitis* gene. When the relationship is one-2-many or many-2-many, a new gene product symbol should be attributed. The new symbol will consist of a root with common protein group term (enzyme, transcription factor, transporter, elicitor family…) paired with a number higher than the highest number used already for both *Vitis* and *Arabidopsis*. Alternatively, as Gramene provides pre-computed alignments and phylogenetic trees, we would recommend to use these and include the new *Vitis* genes, for the sake of uniformity. If a tree has to be generated *de novo*, curators can find useful resources at [35]. It is recommended to use branch support or bootstrapping to validate tree structure. Poorly supported branches, like bootstrap values below 70% should be collapsed, because values below this level imply a potentially misleading hierarchy. The phylogenetic trees are based on alignments that should be calculated from codons (at the nucleotide level) rather than with the amino acid sequences, to increase the discriminative power between closely related *Vitis* genes. Grapevine genes (two or more) at the same phylogenetic distance from a single homolog in *Arabidopsis* should be differentiated by a number. If the Arabidopsis gene name ends with a number, the characters used to differentiate the *Vitis* genes should be letters.

#### Examples of gene name confusion and the recommended nomenclature procedure

**Figure 4:**
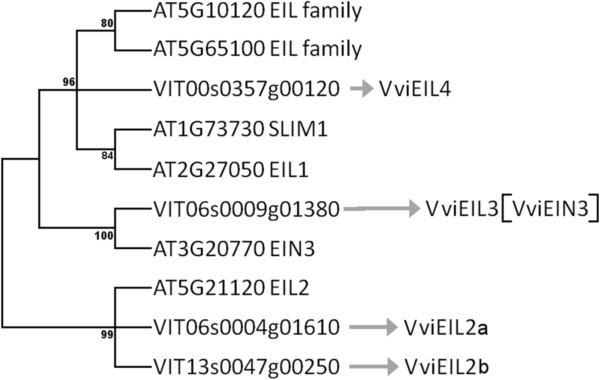
**Molecular phylogenetic analysis of** ***Vitis vinifera*** **and** ***Arabidopsis thaliana*** **EIL gene models by the maximum likelihood method.** Multiple sequence alignment for full-length transcription factors was inferred using MUSCLE [36]. The evolutionary history was inferred by using the Maximum Likelihood method based on the JTT matrix-based model [37]. The bootstrap consensus tree inferred from 100 replicates [38] is taken to represent the evolutionary history of the taxa analyzed [38]. Branches corresponding to partitions reproduced in less than 70% of bootstrap replicates were collapsed. The percentage of replicate trees in which the associated taxa clustered together in the bootstrap test (100 replicates) is shown next to the branches [38]. Initial tree(s) for the heuristic search were obtained automatically by applying Neighbor-Join and BioNJ algorithms to a matrix of pairwise distances estimated using a JTT model, and then selecting the topology with superior log likelihood value. The analysis involved 10 amino acid sequences. The coding data was translated assuming a Standard genetic code table. All positions containing gaps and missing data were eliminated. There were a total of 273 positions in the final dataset. Evolutionary analyses were conducted in MEGA5 [39]. Arrows point toward recommended *Vitis* symbols.

To highlight different gene name problems and the recommended resolution, four examples are described in the following section:

*Example 1. Uncharacterized members in Arabidopsis and members with diverse names: the EIL family (Figure*4*).*

The four *Vitis* genes that have been identified as *EIN3*-like transcription factors (*EIL*) [7] were compared to the *EIL* genes of *Arabidopsis* found in the plantTFDB [40] and a phylogenetic tree was reconstructed. Plant transcription factor family symbols are available in plantTFDB or plnTFDB [41] and can be used for comparison with *Vitis*.

The gene *VIT06s0009g01380* is orthologous to *Arabidopsis EIN3*. Even though *EIN3* is the gene that gives its name to the whole family, it does not conform to the family name symbol and refers to a phenotype. In addition, there is no evidence that the grapevine gene induces the *EIN3* phenotype. Under these circumstances it is recommended to name the *Vitis* ortholog *EIL3*, because the number 3 is the next available numbers used for *Arabidopsis.* The symbol *VviEIN3* would then be used as a synonym. The choice of the lead symbol and the synonym should be left to the curator’s discretion since it will depend on the history of the gene and additional evidences on the function (or phenotype). Only in the case that a similar function or phenotype, described for an *Arabidopsis* gene, could be experimentally demonstrated in *Vitis*, then only the name *EIN3* would be justified. In any other case *EIL3* should be favored.

Two genes are equally distant from *EIL2*. Since there are two genes, an additional letter should follow the symbol to differentiate them.

The last *Vitis* gene *VIT00s0357g00120* is equidistant from two unnamed and unclassified *EIL*s, and from *SLIM1* and *EIL1*. Therefore, the root will be ‘EIL’ and the index, the next available independent number. To avoid any confusion, the recommended symbol under these conditions should be *VviEIL4*.

There is no order in which *VIT06s0009g01380* and *VIT00s0357g00120* should be named; either one can be *VviEIL3* or *VviEIL4*.

*Example 2. Genes already named in grapevine, but names inconsistent with Arabidopsis and Arabidopsis genes without symbols: sugar transporters.*

The grapevine sugar transporter genes were classified by Afoufa-Bastien et al. [3]; when available, their classification was based on the literature. Three of the sugar transporter families provide examples for different scenarios.

**Figure 5:**
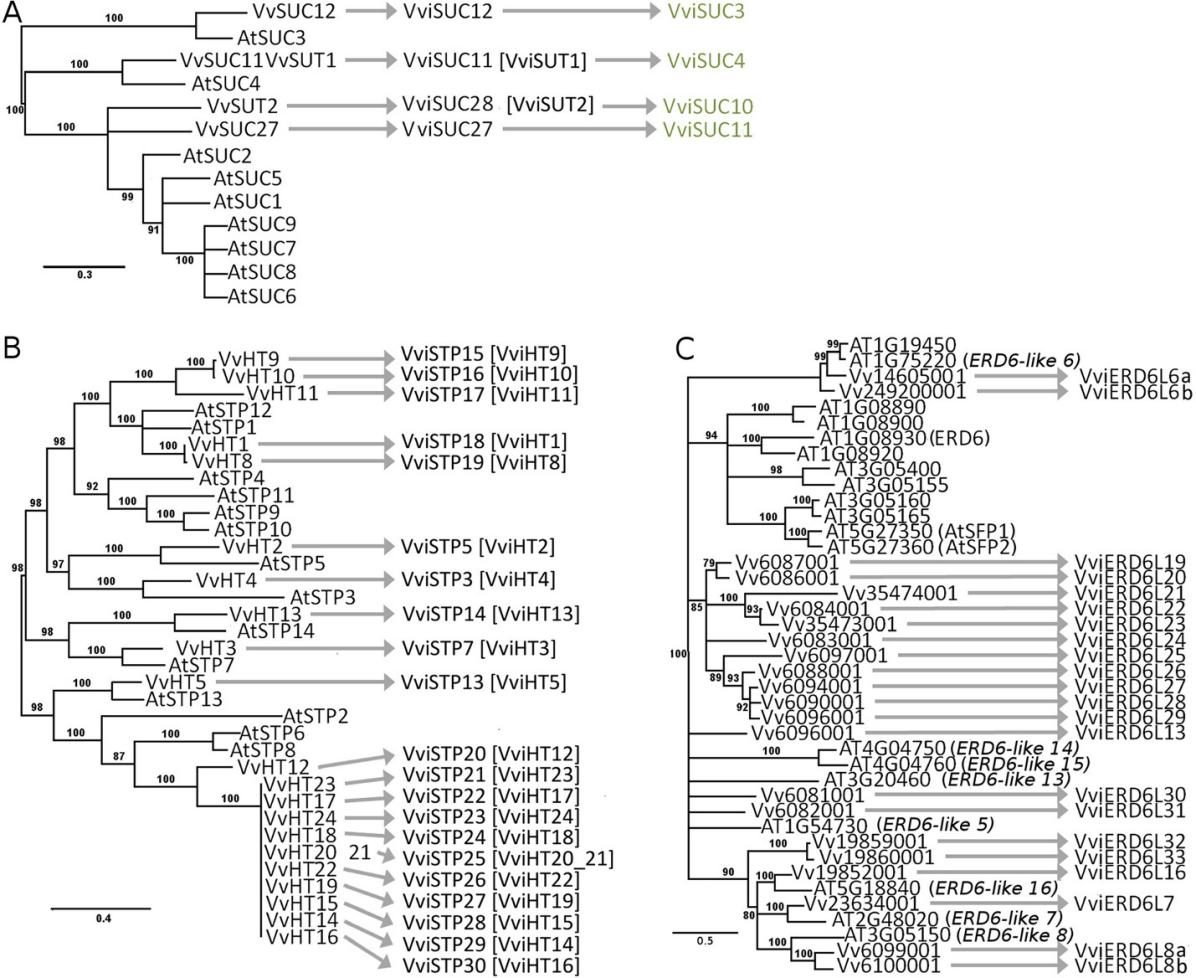
**Molecular phylogenetic analysis of** ***Vitis vinifera and Arabidopsis thaliana*** **sugar transporter gene models by the Maximum Likelihood method.** The trees are adapted from [3] and produced using MUSCLE [36] and PhyML with the JTT amino acid substitution model. Bootstrapping was performed with 100 replicates. In addition to the original picture, branches corresponding to partitions reproduced in less than 70% of bootstrap replicates were collapsed. The percentage of replicate trees in which the associated taxa clustered together in the bootstrap test (100 replicates) is shown next to the branches [38]. **A)** sucrose transporters **B)** hexose transporters **C)** ERD6-like proteins. Arrows point toward recommended *Vitis* symbols, the green symbols are the putative symbols that would be used had not the *Vitis* gene been previously annotated in the literature. Recommended synonyms are in brackets.

The sucrose transporter family was classified by Davies et al. [42] as *SUCXX* and by Ageorges et al. [43] as *SUTXX* with the *SUC11/SUT1* gene being identified and named differently in the two papers. The phylogenetic tree drawn by [3] (adapted in Figure 5A) shows the genetic distance with the *Arabidopsis* genes and the proposed names of the symbols are shown in the middle column where the *SUCXX* format is prioritized as in *Arabidopsis*; as shown here *SUT1* should be used as a synonym for *SUC11. SUT2* should be kept as a synonym and a new name fitting the “SUC” format needs to be created. Since there is no closest ortholog, the number should be incremented after the highest number in both *Vitis* and *Arabidopsis*, which is *VviSUC28*. The names that would have been used if the genes were not named in earlier publications and only theoretically inferred by homology are indicated in the right section of Figure 5A.

The grapevine hexose transporters were symbolized as *HTXX* and functionally characterized [44] for *HT1*, [45] for *HT3*, *HT4*, *HT5*. Other sequences were identified and classified up to *HT24* [3]. However, in *Arabidopsis* this family is named sugar transporter proteins (*STP*). As a consequence, it is recommended that the symbols under the *VviHTXX* format should be kept as synonyms and the main symbol should be under the *VviSTPXX* format; the numbering of the genes should be in accordance with the phylogenetic tree performed in [3] as adapted in Figure 5B.

The grapevine sugar transporter *ERD6*-like family was also compared to *Arabidopsis* [3]; the phylogenetic tree was adapted in Figure 5C. In this work, no symbols were assigned to the *Arabidopsis* genes, probably because they were never published, even though a nomenclature existed and they appeared as full names in the UniProt and NCBI databases. As a consequence no symbols were transferred to the *Vitis* genes in that publication. In addition, since the symbol *ERD6* ends with a number it is recommended to add the letter L, for -like, between the family root of the symbol and the number as presented in Figure 5C. This family in *Vitis* contains also a branch that is not related to *Arabidopsis;* the numbers of the genes in this branch shall be incremented after the last known number for the *Arabidopsis* genes.

*Example 3. When gene name and function change with new discoveries: the CCD family and the NCED subfamilies.*

**Figure 6:**
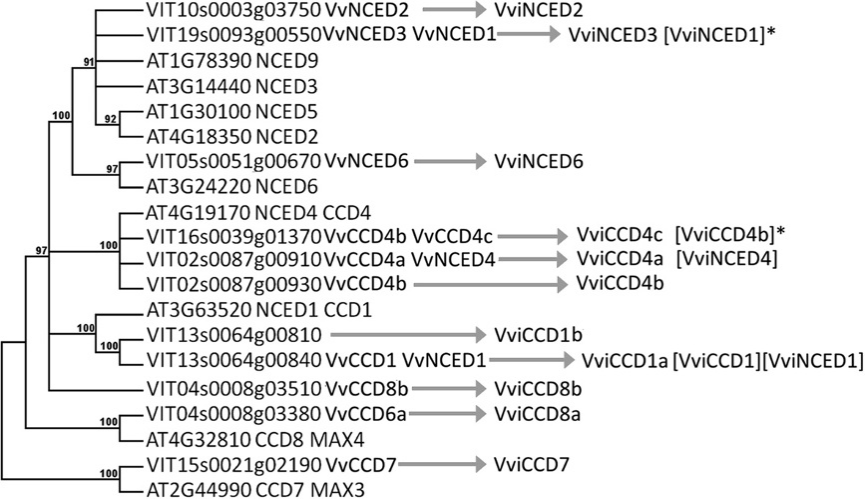
**Molecular phylogenetic analysis of** ***Vitis vinifera and Arabidopsis CCD and NCED*** **gene models by the Maximum Likelihood method.** Multiple sequence alignment for full-length carotenoid cleavage dioxygenases was inferred using MUSCLE [36]. The evolutionary history was inferred by using the Maximum Likelihood method based on the JTT matrix-based model [37]. The bootstrap consensus tree inferred from 100 replicates [38] is taken to represent the evolutionary history of the taxa analyzed [38]. Branches corresponding to partitions reproduced in less than 70% of bootstrap replicates were collapsed. The percentage of replicate trees in which the associated taxa clustered together in the bootstrap test (100 replicates) is shown next to the branches [38]. Initial tree(s) for the heuristic search were obtained automatically by applying Neighbor-Join and BioNJ algorithms to a matrix of pairwise distances estimated using a JTT model, and then selecting the topology with superior log likelihood value. The analysis involved 20 amino acid sequences. The coding data was translated assuming a Standard genetic code table. All positions containing gaps and missing data were eliminated. There were a total of 225 positions in the final dataset. Evolutionary analyses were conducted in MEGA5 [39]. Arrows point toward recommended *Vitis* symbols. Asterisks indicate redundant synonyms.

The *Vitis* genes for the *CCD/NCED* family were characterized and named according to homology with genes from *Arabidopsis* [41, 42], although some were characterized in previous studies. The phylogenetic tree was independently rebuilt in Figure 6 and differs from the one presented in [46] since genes from non-*Arabidopsis* species were used. The tree is similar to [47] except for the genes not present in that study. Three previously undetected genes were added (*VviCCD8b* in [47] and *VviCCD4b VviCCD1b* in [46]), but the gene’s nomenclature would have been relatively similar. The *NCED* genes are a subset of the *CCD* family and they share similar features, including sequence similarity and carotenoid double-bond-cleaving dioxygenase activity. *CCD*s are distinguished by the specificity of double bond cleavage and *NCED’s* are plastid-localized [48]. Hereby, the genes belonging to the *NCED* family should only bear the *NCED* symbol, likewise for the *CCD* genes, to avoid confusion. However, two historical members were named *CCD1/NCED1*, and *CCD4/NCED4*. In this case both symbols should be kept with *CCD1* (or 4) as the main symbol and *NCED1* (or 4) as the synonym, since this gene presents a more *CCD*-like function as demonstrated in [47]. A note should be linked to the *NCED* synonym to indicate its obsolescence.

Since a second gene from grapevine appears to belong to the *CCD1* subgroup, the genes should be renamed with an extra character to differentiate them (*CCD1_1 and CCD1b*); however the symbols “*CCD1*” and “*NCED1*” were attributed to *CCD1a* and should be kept as synonyms for it. Since *VviCCD4b* was not identified in [47], authors named *VviCCD4c* with the letter b and [46] also named *VviCCD4b* with the letter b. To avoid any kind of confusion, new names can also be allocated to these genes and all the previous names should be reported as synonyms with a note indicating that a given synonym has been used for multiple genes.

Similarly, *VviNCED3* was incorrectly identified as *NCED1* in [49]. Therefore, *VviNCED1* should appear as a *VviNCED3* synonym but with a note indicating that this synonym is incorrect.

The gene *VIT04s0008g03510*, coding for a member of the well described *CCD8b* group of orthologous genes in the grapevine was named with this symbol even though no *Arabidopsis* gene belongs to this family, because it is a well described group of orthologous genes [46].

*Example 4. Genes not present in Arabidopsis: the STS family.*

**Figure 7:**
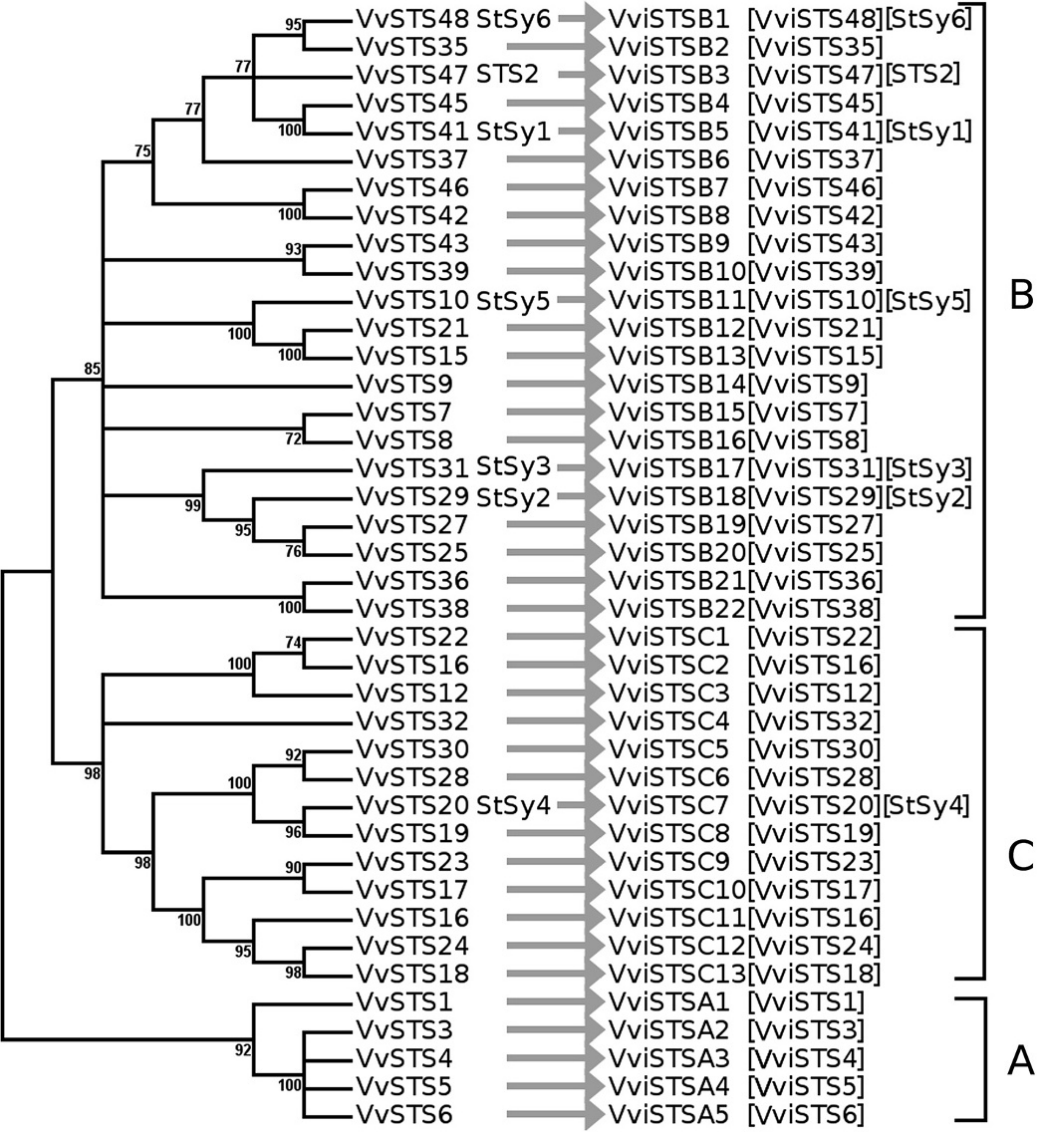
**Molecular phylogenetic analysis of** ***Vitis vinifera*** **trihydroxystilbene synthase gene models by the Maximum Likelihood method.** Multiple sequence alignment for full-length trihydroxystilbene synthases was inferred using MUSCLE [36] from the nucleotide sequence. The evolutionary history was inferred by using the Maximum Likelihood method based on the JTT matrix-based model [37]. The bootstrap consensus tree inferred from 100 replicates [38] is taken to represent the evolutionary history of the taxa analyzed [38]. Branches corresponding to partitions reproduced in less than 70% of bootstrap replicates were collapsed. The percentage of replicate trees in which the associated taxa clustered together in the bootstrap test (100 replicates) is shown next to the branches [38]. Initial tree(s) for the heuristic search were obtained automatically by applying Neighbor-Join and BioNJ algorithms to a matrix of pairwise distances estimated using a JTT model, and then selecting the topology with superior log likelihood value. The analysis involved 40 amino acid sequences. The coding data was translated assuming a standard genetic code table. All positions with less than 95% site coverage were eliminated. That is, fewer than 5% alignment gaps, missing data, and ambiguous bases were allowed at any position. There were a total of 292 positions in the final dataset. Evolutionary analyses were conducted in MEGA5 [39]. Arrows point toward recommended *Vitis* symbols. **A,B,C** refer to the groups in [4].

The grapevine trihydroxystilbene synthase (STS) gene family was characterized in two concomitant articles [4, 5]. As this family is not present in *Arabidopsis*, it is not possible to rely on sequence similarity with the *Arabidopsis* genes to address the nomenclature. While describing the genes, both authors used the same strategy to name the genes according to the syntenic positions, which is logical since the genes are grouped in two clusters on chromosomes 10 and 16. The names in both studies are identical. However, some of the genes were already described in previous studies [50, 51], and this was not taken into account for the naming of the members of the STS family. The genes were stored in public databases such as UniProt and Refseq under their original denominations. The symbols are written differently, STS vs StSy, while the full names are both trihydroxystilbene synthase. This causes problems: for example, trihydroxystilbene synthase 5 may refer to two different genes (*Stsy5/VvSTS10* and *VvSTS5*); thus, the symbols are distinct but the full names are identical. There was one gene, however, (*VvSTS47*), that was previously named with an *STS*-like symbol (*STS2*) in addition to the synonyms (*VINST1*, *PSV25*, *VST1*). There is no problem in keeping *VvSTS47* as a synonym, but the symbol *STS2* refers to two different genes (*VvSTS2* and *VvSTS47*) which causes confusion. The strategy of ordering according to the chromosome position should be avoided. It presents the disadvantage of being invalidated each time changes occur at the level of the genome assembly or when new members of the family are discovered. It is therefore recommended to conserve the phylogenetic tree strategy for gene naming (Figure 7).

### Annotation platform and informatics tools

There is a need for a centralized online platform that allows manual curation of gene-models and their functional annotation by experts. Besides the central repository, several other (offline) resources are available that can be used to improve the annotation.

#### Platform for community curation of grapevine gene annotation

The annotation platform for the grapevine genome is centralized and maintained in the ORCAE database with online interface from the VIB [25] and was chosen to perform community annotation for *Vitis*. ORCAE was developed with a gene-centric vision, meaning that the gene information pages are the central access points instead of a genome browser. The basic setup of ORCAE can be compared to a wiki system with information pages for each gene like a ‘topic’ page of a traditional wiki text. ORCAE was designed to suit the needs of genome sequencing projects from small consortia, like the grapevine. Like wikis, the data stored in ORCAE is never removed and a complete history of the changes applied by curators is kept. Also a number of analyses are run and updated in the background after changes affecting the gene structures have been supplied. Updates to central repositories, like NCBI, will be organized on a six months basis, if the number of modifications can be considered as worthwhile. Users, willing to manually curate data will have to register with the ORCAE system, mostly to allow communication between curators worldwide. Also accounts are a way to remediate when erroneous modifications occur or to track errors in the input data, and discuss with the authors that mistakenly entered incorrect data. The whole systems history of modifications allows the retrieval of previous versions of gene models. Furthermore, to limit simple errors, tests have been implemented for checking the editing process, via the GenomeView application. These checks result in the ability of the system to reject genes models that contain obvious errors after user’s modifications. Genes that would be missing from the current genome assembly, but are proven to be in Vitis, will be added to ORCAE as standalone genes, although, only after thorough checking to ensure that they are actually real. As for the genes represented in the reference genome, they will follow the same process for submitting annotation to NCBI and their nomenclature will follow the same rules as for other genes.

#### Guidelines for community gene functional and structural annotation

The sNCGGa can be contacted from the IGGP website at http://www.vitaceae.org/index.php/Annotation. Official announcement from the committee can be found at that address. A preliminary functional annotation tutorial is also available [52] and will be updated with the present paper. Topics described in this tutorial are open to debate and can be amended during the process of community annotation. The sNCGGa can be contacted for enquiries at the Google group.

One of the major goals is to bring together experts for each gene family to allow them to perform their annotation through the ORCAE annotation website, which in due time will synchronized with major public databases such as NCBI or Uniprot. The annotation should fit the IGGP Committee guidelines in terms of nomenclature and rules for addressing the level of confidence. In any case where possible, it is advised to annotate complete gene families or all the enzymes involved in a metabolic pathway, rather than a single isolated member of a larger group of genes.

## Conclusions

The intent of the grapevine nomenclature standardization is, taking into account the accumulated experience from other species and in grapevine, to clear up gene name confusion and redundancy. In particular we want to anticipate on the ever-growing amount of new sequencing data. It is important to consider that the collection of experimental evidence for grapevine genes will most likely be limited and that the community is forced to opt for a strategy that can consider annotation inferred from similarity to other species. This is a problem endemic to small and medium-sized research communities. With the current paper, it was chosen to propose a set of guidelines aiming at a harmonized nomenclature for the full names and symbols of *Vitis* genes that allow easy correspondence with other species, without being restrictive or too rigid. On the other hand the attribution of the locus ID is done automatically and will be systematically attributed to each new gene according to internal rules. This rule-based nomenclature system is intended to reduce confusion, improve gene and protein comparisons, and facilitate the comparison of functions across species. The success of a nomenclature system requires the participation of the grape community, who by contributing will share the knowledge through discussions and through implementation of the system to improve grape gene nomenclature and annotation.

## Methods

### Phylogenetic analysis

Multiple sequence alignment was inferred using MUSCLE [36]. The evolutionary history was inferred by using the Maximum Likelihood method based on the JTT matrix-based model [37]. The bootstrap consensus tree inferred from 100 replicates [38] is taken to represent the evolutionary history of the taxa analyzed [38]. Branches corresponding to partitions reproduced in less than 70% of bootstrap replicates were collapsed. Initial tree(s) for the heuristic search were obtained automatically by applying Neighbor-Join and BioNJ algorithms to a matrix of pairwise distances estimated using a JTT model, and then selecting the topology with superior log likelihood value. The coding data was translated assuming a Standard genetic code table. All positions containing gaps and missing data were eliminated.
